# Detection of aberrant promoter methylation of RNF180, DAPK1 and SFRP2 in plasma DNA of patients with gastric cancer

**DOI:** 10.3892/ol.2014.2410

**Published:** 2014-08-04

**Authors:** XIE ZHANG, XUESONG ZHANG, BEILEI SUN, HONGNA LU, DANPING WANG, XIAOGANG YUAN, ZHIGANG HUANG

**Affiliations:** 1 Department of Gastroenterology, Ningbo Medical Treatment Center, Li Huili Hospital, Ningbo, Zhejiang 315040, P.R. China; 2 School of Medicine, Ningbo University, Ningbo, Zhejiang 315211, P.R. China

**Keywords:** gastric cancer, RNF180, DAPK1, SFRP2, hypermethylation, methylation-specific polymerase chain reaction

## Abstract

Gastric cancer (GC) is one of the most frequently diagnosed malignancies in East Asia, particularly in China, and remains the second leading cause of cancer-associated mortality worldwide. However, no effective plasma biomarkers have been identified for the diagnosis of patients with GC. The aim of this study was to investigate the DNA methylation status of the ring finger protein 180 (RNF180), secreted frizzled-related protein 2 (SFRP2) and death-associated protein kinase 1 (DAPK1) genes in the plasma samples of 57 GC patients and 42 control individuals with no malignant disease, and to evaluate the clinical utility of these makers. A significantly higher level of methylation was observed in the plasma DNA of GC patients when compared with that of controls for the three genes investigated (RNF180, 57.89% vs. 23.81%; DAPK1, 49.12% vs. 28.57%; and SFRP2, 71.93% vs. 42.86%). No association was identified between the DAPK1 or SFRP2 methylation level in the plasma DNA and the clinicopathological parameters of patients. Notably, RNF180 methylation was found to positively correlate with tumor size (P=0.018), histological type (P=0.025), TNM stage (P=0.002), lymph node metastasis (P=0.008) and distant metastasis (P=0.018). Overall, 50 cancer patients (87.72%) exhibited methylation of at least one of the three markers, while 26 normal subjects presented methylation in plasma DNA [specificity, 38.1%; odds ratio (OR), 4.4]. The combined use of RNF180 and SFRP2 as methylation markers appeared to be the most preferable predictor with regard to predictive power and cost-performance (OR, 5.57; P=0.0002). The results of the present study indicate that aberrant promoter methylation of genes in the plasma may be detected in a substantial proportion of GC patients and thus, these genes must be evaluated in the screening and surveillance of GC.

## Introduction

Gastric cancer (GC) is one of the most frequently diagnosed malignancies in East Asia, particularly in China (1,2). Detection of the disease in the preclinical or pre-symptomatic phases is the key to successful treatment and patient outcome. In China, gastroscopy is increasingly used as a primary detection tool due to its diagnostic accuracy. However, less than half of eligible individuals (>50 years of age) undergo GC screening. Numerous patients are diagnosed at an advanced stage, leading to a high mortality rate of GC (3). The identification of more reliable and noninvasive screening tests may increase compliance with GC screening guidelines by individuals who are reluctant to undergo invasive tests, or when gastroscopy detection is not feasible or readily available.

A number of studies have demonstrated that cancer patients exhibit significantly higher serum levels of tumor-specific DNA mutations, with >90% of the total circulating cell-free DNA (cfDNA) derived from malignant tissue, compared with those with non-malignant diseases (4–8). The mechanism of cfDNA release into the circulation is poorly understood; however, it is hypothesized that methylation of gene promoter regions is crucial for tumor carcinogenesis (6). These alterations have been shown to occur early in the development of cancer (9), suggesting that the detection of free methylated circulating DNA presents a promising approach for the development of plasma-based screening methods for non-invasive monitoring of GC progression.

The aim of the present study was to evaluate the potential of using the promoter methylation status of a panel of apoptosis-related genes, including the ring finger protein 180 (RNF180) (10), death-associated protein kinase 1 (DAPK1) (11,12) and secreted frizzled-related protein 2 (SFRP2) (11,13) genes, in circulating plasma DNA for the detection of GC. The three genes included in this study were selected as they are known to be functional tumor suppressor genes in GC, and have previously been reported to be highly methylated in tumors and the serum/plasma of GC patients (11–15). The methylation-specific polymerase chain reaction (PCR) (MSP) technique was used to analyze the specificity and sensitivity of this method for GC detection and to evaluate the clinical diagnostic significance of these DNA methylation-based plasma markers.

## Materials and methods

### Patients and samples

A total of 42 controls and 57 GC patients diagnosed at Li Huili Hospital (Ningbo, China) between July 2011 and July 2012 were enrolled in this study. None of the enrolled patients had received preoperative chemotherapy or radiation therapy. This study was approved by the ethics committee of Li Huili Hospital (Ningbo, China) and informed consent was obtained from all participants. All patients were diagnosed with GC based on pathological and/or cytological evidence. The clinicopathological features are shown in Table I. Tumor stage was determined according to the tumor node metastasis (TNM) criteria of the Union for International Cancer Control/American Joint Committee on Cancer, 2010 (16). The plasma samples were acquired prior to treatment in the patient and control groups. The plasma samples were immediately isolated by centrifugation at 1,000 × g for 10 min and stored at −80°C until DNA was extracted.

### DNA extraction and bisulfite modification

A total of 400 μl DNA from each plasma sample was extracted using the QIAamp DNA Blood mini kit (Qiagen, Hilden, Germany)according to the manufacturer’s instructions. Plasma DNA was eluted in a total volume of 80 μl elution buffer (EB) and stored at −20°C.

Sodium bisulfite modification was conducted using the Qiagen Epitect Plus DNA bisulfite kit (Qiagen), according to the manufacturer’s instructions. DNA was then resuspended in 30 μl EB and stored at −20°C.

### MSP

Methylated and unmethylated primers specific for the promoter sequences of the target genes, RNF180, DAPK1 and SFRP2, were designed to amplify the bisulfite-modified DNA. The primer sequences used are shown in Table II. The 50-μl reaction mixture contained 2 μl of DNA template, 10 μl of KAPA2G buffer (Kapa Biosystems, Woburn, MA, USA), 1 μl of 10 mM dNTP mix (Kapa Biosystems), 1 μl of each primer at 50 mM, and 0.5 units of KAPA2G^TM^ Robust Hotstart DNA polymerase (Kapa Biosystems).

The conditions for amplification included a single cycle at 95°C for 5 min and a subsequent 10 cycles at 95°C for 30 sec, melting temperature 8°C (Tm; 0.8°C, descending by 0.8°C for each cycle) for 60 sec and 72°C for 30 sec; then 38 cycles at 95°C for 30 sec, Tm for 60 sec and 72°C for 30 sec; followed by a final extension step of 10 min at 72°C. The PCR products were then electrophoresed on a 2.5% agarose gel and visualized under ultraviolet illumination (ChemiDoc XRS; Bio-Rad, Hercules, CA, USA). Each experiment was repeated at least three times. The operator who performed all assays was blinded to all clinical information.

### Statistical analysis

SPSS, version 13.0 (SPSS, Inc., Chicago, IL, USA) was used for all statistical analyses. The mean of variables was compared between two groups using Student’s t-test. The association between the methylation status of various genes and the clinicopathological characteristics of patients was evaluated using the χ^2^ test or the Fisher’s exact test. To estimate the predictive power of clinical and plasma markers for the presence of GC, multivariate logistic regression analyses were performed. Odds ratios (ORs) with 95% confidence intervals (CIs) were used as a measure of association. P<0.05 was considered to indicate a statistically significant difference.

## Results

### Patient characteristics

A total of 57 GC patients and 42 healthy controls donated blood within a 12-month time period prior to receiving any treatment. The mean age ± standard deviation of patients with GC was 61.49±12.02 years, while that of the controls was 57.21±8.45 years. The ratio of male to female patients was 39:18 in the cancer group and 27:15 in the control group. No significant differences in age and gender were identified between the two groups (data not shown). The clinicopathological characteristics of the 57 GC patients are shown in Table I.

### Detection of aberrant RNF180, DAPK1 and SFRP2 promoter methylation in plasma

**Figure 1:**
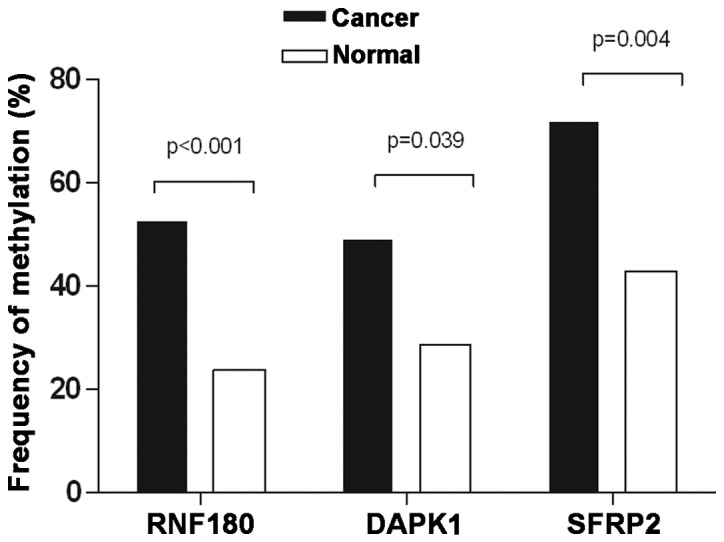
Frequency of detecting methylated DNA in the plasma of gastric cancer patients and controls. RNF180, ring finger protein 180; DAPK1, death-associated protein kinase 1; SFRP2, secreted frizzled-related protein 2.

**Figure 2:**
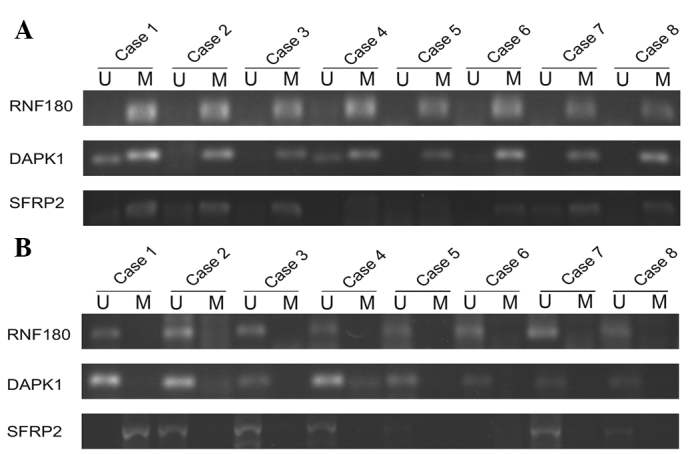
Representative MSP results of RNF180, DAPK1 and SFRP2 aberrant methylation in (A) gastric cancer and (B) control patients. U, results obtained using unmethylated primers; M, results obtained using methylated primers; MSP, methylation-specific polymerase chain reaction; RNF180, ring finger protein 180; DAPK1, death-associated protein kinase 1; SFRP2, secreted frizzled-related protein 2.

To determine whether the DNA methylation status of the RNF180, DAPK1 and SFRP2 genes in plasma samples had diagnostic value for GC, MSP analysis was used to investigate the frequency of DNA methylation of these genes in the plasma samples of 42 control and 57 GC patients. In the peripheral blood plasma, RNF180 methylation was detected in 57.89% (33/57) of GC patients, while methylation of this gene was observed in 23.81% (10/42) of noncancerous control patients (P=0.0007; Fig. 1). The methylation frequencies of DAPK1 were 49.12% (28/57) in GC patients and 28.57% (12/42) in noncancerous controls (P=0.0394; Fig. 1). Regarding SFRP2, methylation was detected in 71.93% (41/57) of GC patients and 42.86% (18/42) of control patients (P=0.0036; Fig. 1). Representative agarose gel electrophoresis results of the MSP for the three genes are shown in Fig. 2.

### Association between promoter methylation in plasma DNA and clinicopathological parameters of GC patients

The clinicopathological characteristics of the GC patients and the methylation status of RNF180, DAPK1 and SFRP2 are shown in Table I. No association between DAPK1 and SFRP2 methylation in the plasma DNA, and gender, age, tumor size, differentiation status, TNM stage, lymph node metastasis or distant metastasis were identified. However, the methylation levels of the RNF180 gene were found to positively correlate with tumor size (P=0.018), histological type (P=0.025), TNM stage (P=0.002), lymph node metastasis (P=0.008) and distant metastasis (P=0.018).

### Comparison of the predictive power of RNF180, DAPK1 and SFRP2 methylation and their combination for GC detection

Multivariate regression analyses revealed a significant correlation between GC and RNF180 methylation (OR, 3.528; 95% CI, 0.542–0.861; P=0.007) and SFRP2 methylation (OR, 2.647; 95% CI, 1.080–6.487; P=0.033), but not for DAPK1 methylation (OR, 1.540; 95% CI, 0.610–3.890; P=0.361), in GC patients and controls (Table III).

The ORs (95% CI) for predicting the presence of GC using the RNF180 (P=0.0007), DAPK1 (P=0.0394) and SFRP2 methylation statuses (P=0.0036) were 4.40 (1.82–10.65), 2.41 (1.03–5.63) and 3.42 (1.47–7.92), respectively (Table IV). Furthermore, if RNF180 and DAPK1 methylation were combined, the OR (95% CI) for cancer prediction [OR, 4.86 (2.03–11.66); P=0.0003] was similar to that of the RNF180 methylation [OR, 4.40 (1.82–10.65); P=0.0007] alone and to that of the methylation of the three genes combined [OR, 4.40 (1.61–12.03); P=0.0026]. When RNF180 and SFRP2 methylation were combined, the OR (95% CI) for cancer prediction [OR, 5.57 (2.13–14.57); P=0.0002] was superior to that for RNF180 methylation alone [OR, 4.40 (1.82–10.65); P=0.0007]; however, the specificity (47.62%) for cancer prediction was markedly lower than that of RNF180 methylation alone (76.19%).

Overall, the combination of RNF180 and SFRP2 methylation appeared to be the most effective predictor of GC, with regard to predictive power and cost-performance.

## Discussion

Aberrant promoter methylation is the predominant mechanism which inactivates tumor-associated genes, particularly tumor suppressor genes, along with genetic silencing, which ultimately leads to gastric carcinogenesis. Numerous genes have been found to be methylated in GC, including hMLH1, p16, RUNX3, DAPK1, SFRP2 and RNF180. hMLH1 encodes DNA repair proteins and is closely associated with poor prognosis of GC patients; the frequency and specificity were found to be 8.6–80 and 4.1–80%, respectively (17–19). P16 inhibits cell cycle progression, and has been found to correlate with poor tumor differentiation, lymph node metastasis and poor survival. The frequency and specificity of P16 were found to be 30.4–44.2 and 76–100%, respectively (15,18,20). RUNX3 belongs to the RUNX family of transcriptional factors, and has been found to correlate with depth of tumor invasion, lymph node and distant metastasis. The frequency and specificity of RUNX3 were 56–75.2 and 92.6%, respectively (21–23). DAPK1 is a positive regulator of cell apoptosis, and has been found to correlate with poorly differentiated tumors and lymph node metastasis. The frequency and specificity of DAPK1 were 30.9–83.2 and 57.8–100%, respectively (12,15,24–26). No significant correlation has been identified between SFRP2, a candidate tumor-suppressor gene, and clinical outcomes. The frequency and specificity of SFRP2 were 80.0–90.0 and 31.0–73.3%, respectively (13,27). RNF180 is a novel potential tumor suppressor in GC; however, no correlation with clinical outcomes was identified. The frequency and specificity of RNF180 were 76% and 100%, respectively (10).

However, our preliminary study revealed that the methylation of hMLH1, P16 and RUNX3 plasma biomarkers was extremely low in GC patients (data not shown). Of note, in the current study, the methylation levels of the apoptosis-related genes, RNF180, DAPK1 and SFRP2, were observed to be significantly higher in the plasma DNA of GC patients when compared with controls.

The methylation of a core functional region of the promoter of RNF180, a novel ring finger-encoded product, has been suggested to significantly correlate with human GC development and pre-cancerous lesions. RNF180 acts as a potential tumor suppressor, exhibiting a critical role in the suppression of cell proliferation and induction of apoptosis (27). Cheung *et al* (10) demonstrated that methylation of RNF180 was detected in 56.25% (18/32) of plasma samples from cancer patients, whereas RNF180 methylation was not detected in the plasma of 64 normal controls. In the present study, the results revealed that the methylation of RNF180 was detected in 57.89% (33/57) of GC patient plasma samples and in 23.81% (10/42) of the controls. The frequency of RNF180 methylation was 23.81% (10/42) in controls. Although the specificity was lower, the GC patient population tested in the current study was larger than that of the previous report (10). Additionally, in the present study, the association of plasma DNA methylation of RNF180 in each sample with clinical outcomes was investigated.

DAPK1 is a calcium/calmodulin-dependent serine/threonine protein kinase involved in apoptosis and tumor suppression (14,15). Reduced expression and aberrant methylation of DAPK1 has been reported in numerous human cancers, including GC (28,29). In previous studies, the sensitivity and specificity of predicting GC using the serum DNA methylation status of DAPK1 were found to be 48.1% (26/54) and 100% (0/30), respectively (12). However, in the present study, DAPK1 plasma methylation was detected in 49.12% (28/57) of GC patient, and in 28.57% (12/42) of the controls. Although the sensitivity was higher than previously reported, the specificity remained low.

SFRP2 has been identified as a modulator of the Wnt signaling pathway, which is associated with multiple tumor types, including GC (13,31). Recent epigenetic studies have demonstrated that silencing of the SFRP2 by promoter methylation at CpG islands enhanced tumor growth and expansion in GC (13,32,33). Cheng *et al* (13) revealed that serum SFRP2 methylation was a potential biomarker for GC, as SFRP2 methylation was detected in a total of 66.7% (12/18) GC patients, however, no SFRP2 methylation was detected in the sera of 18 normal subjects. Similarly, in the current study, SFRP2 methylation was detected in 71.93% of GC patient plasma samples, however, the specificity of this single biomarker was relatively low (57.14%).

Considering that aberrant DNA methylation of plasma exhibits a significant role in GC development and progression, the clinical significance of DNA methylation was investigated to evaluate the diagnostic power of the RNF180, DAPK1 and SFRP2 markers in plasma. Firstly, the association between the plasma DNA methylation status of RNF180 in each sample and clinical data was evaluated. Of note, tumor size (P=0.018), histological type (P=0.025), TNM stage (P=0.002), lymph node metastasis (P=0.008) and distant metastasis (P=0.018) were found to be significantly associated with RNF180 methylation, indicating that this epigenetic alteration may be a valuable marker for the prognosis of GC patients. However, no significant correlation was identified between DAPK1 and SFRP2 methylation in the plasma DNA and the clinicopathological characteristics evaluated, indicating that these epigenetic events are involved in the multistep process of gastric carcinogenesis, and may present potential biomarkers for early diagnosis in GC. Further studies may be required to characterize the source of cfDNA and the mechanisms involved in its release into the blood to provide an improved explanation for this observation.

One strategy to improve GC diagnosis is to combine multiple methylation biomarkers in plasma that have potential clinical applications. In particular, the high specificity of RNF180 methylation is an attractive candidate as a marker for such a panel, which would exhibit increased sensitivity without major impacts on specificity. The combination of RNF180, DAPK1 and SFRP2 methylation in this study did increase sensitivity, but at a cost to specificity. Thus, a pilot study investigating the performance of RNF180 methylation in combination with SFRP2 was carried out. As expected, the combination of RNF180 and SFRP2 methylation exhibited a significantly higher OR and a marginal reduction in diagnostic sensitivity, as well as a higher specificity, when compared with the combination of RNF180, DAPK1 and SFRP2 methylation. Therefore, the combination of RNF180 and SFRP2 methylation may be optimal for GC diagnosis.

In conclusion, using an MSP approach, RNF180 was identified as a novel plasma hypermethylated gene in GC. Although the functional effect of RNF180 methylation in GC was not identified, its high potential as a biomarker in plasma-based DNA testing was demonstrated. Furthermore, this study suggests that combining RNF180 and SFRP2 methylation may be particularly promising. The full potential of this marker or its combination with SFRP2 requires validation in a larger, well-controlled cohort study to verify its performance in detecting GC, and to investigate the potential clinical application for monitoring GC treatment and predicting responses to chemotherapy and radiotherapy.

## Figures and Tables

**Table I:** Clinicopathological features and RNF180, DAPK1 and SFRP2 DNA methylation status in plasma samples of 57 patients with gastric cancer.

		RNF180			DAPK1			SFRP2		
				
Parameters	n	M	U	P-value	M	U	P-value	M	U	P-value
Gender				0.137			0.928			0.446
Male	39	20	15		19	20		27	12	
Female	18	13	9		9	9		14	4	
Age, years				0.627			0.910			0.569
<60	24	13	11		12	12		16	8	
≥60	33	20	13		16	17		25	8	
Tumor size, cm^3^				0.018			0.705			0.784
<6	23	9	14		12	11		17	6	
≥6	34	24	10		16	18		24	10	
Histological type				0.025			0.940			0.769
Differentiated	14	4	10		7	7		10	4	
Undifferentiated	43	29	14		21	22		31	12	
TNM stage				0.002			0.647			0.683
I–II	20	6	14		9	11		15	5	
III–IV	37	27	10		19	18		26	11	
Lymph node metastasis				0.008			0.672			0.660
N0	24	9	15		11	13		18	6	
N1–3	33	24	9		17	16		23	10	
Distant metastasis				0.018			0.961			0.882
M0	50	26	24		24	26		35	15	
M1	7	7	0		4	3		6	1	

RNF180, ring finger protein 180; DAPK1, death-associated protein kinase 1; SFRP2, secreted frizzled-related protein 2; TNM, tumor node metastasis; M, methylated; U, unmethylated.

**Table II:** Primer sequences and annealing temperatures used for the methylation-specific polymerase chain reaction.

Primer	Sequence, 5′-3′	Annealing temperature, °C	Product size, bp	Reference
RNF180 MF	GGAGAAAAATTTTTTTACGGTTTC	50	109	
RNF180 MR	CACGTCTACGAATTCCCAC			
RNF180 UF	AGGGAGAAAAATTTTTTTATGGTTTT	46	109	
RNF180 UR	CACATCTACAAATTCCCACCC			
DAPK1 MF	GGATAGTCGGATCGAGTTAACGTC	52	98	(14)
DAPK1 MR	CCCTCCCAAACGCCGA			
DAPK1 UF	GGAGGATAGTTGGATTGAGTTAATGTT	56	106	
DAPK1 UR	CAAATCCCTCCCAAACACCAA			
SFRP2 MF	GGGTCGGAGTTTTTCGGAGTTGCGC	58	138	(13)
SFRP2 MR	CCGCTCTCTTCGCTAAATACGACTCG			
SFRP2 UF	TTTTGGGTTGGAGTTTTTTGGAGTTGTGT	54	145	
SFRP2 UR	AACCCACTCTCTTCACTAAATACAACTCA			

RNF180, ring finger protein 180; DAPK1, death-associated protein kinase 1; SFRP2, secreted frizzled-related protein 2; MF, methylated forward; MR, methylated reverse; UF, unmethylated forward, UR, unmethylated reverse; bp, base pair.

**Table III:** Multivariate regression analysis for methylation in gastric cancer patients and controls.

Gene methylation	Odds ratio (95% confidence interval)	P-value
RNF180	3.528 (0.542–0.861)	0.007
DAPK1	1.540 (0.610–3.890)	0.361
SFRP2	2.647 (1.080–6.487)	0.033

RNF180, ring finger protein 180; DAPK1, death-associated protein kinase 1; SFRP2, secreted frizzled-related protein 2.

**Table IV:** Comparison of the predictive powers for gastric cancer between RNF180, DAPK1 and SFRP2 methylation alone and in combination.

Gene methylation	Sensitivity, % (95% CI)	Specificity, % (95% CI)	Odds ratio (95% CI)	P-value
RNF180	57.89 (44.08–70.86)	76.19 (60.55–87.95)	4.40 (1.82–10.65)	0.0007
DAPK1	49.12 (35.63–62.71)	71.43 (55.42–84.28)	2.41 (1.03–5.633)	0.0394
SFRP2	71.93 (58.46–83.03)	57.14 (40.96–72.28)	3.42 (1.47–7.92)	0.0036
RNF180 + DAPK1	76.79 (63.58–87.02)	59.52 (43.28–74.37)	4.86 (2.03–11.66)	0.0003
RNF180 + SFRP2	85.96 (74.21–93.74)	47.62 (32.00–63.58)	5.57 (2.13–14.57)	0.0002
DAPK1 + SFRP2	82.46 (70.09–91.25)	42.86 (27.72–59.04)	3.53 (1.41–8.81)	0.0057
RNF180 + DAPK1 + SFRP2	87.72 (76.32–94.92)	38.10 (23.57–54.36)	4.40 (1.61–12.03)	0.0026

RNF180, ring finger protein 180; DAPK1, death-associated protein kinase 1; SFRP2, secreted frizzled-related protein 2; CI, confidence interval.
